# Targeting the mDia Formin-Assembled Cytoskeleton Is an Effective Anti-Invasion Strategy in Adult High-Grade Glioma Patient-Derived Neurospheres

**DOI:** 10.3390/cancers11030392

**Published:** 2019-03-20

**Authors:** Krista M. Pettee, Kathryn N. Becker, Arthur S. Alberts, Kevin A. Reinard, Jason L. Schroeder, Kathryn M. Eisenmann

**Affiliations:** 1 Department of Cancer Biology, University of Toledo Health Science Campus, Toledo, OH 43614, USA; Krista.pettee@utoledo.edu (K.M.P.); Kathryn.becker@rockets.utoledo.edu (K.N.B.); 2 Laboratory of Cell Structure and Signal Integration, Van Andel Research Institute, Grand Rapids, MI 49503, USA; art.alberts@vai.org; 3 Department of Neurosurgery, ProMedica Hospital, Toledo, OH 43606, USA; Kevin.ReinardMD@promedica.org (K.A.R.); jason.schroeder5@utoledo.edu (J.L.S.); 4 Division of Neurosurgery, Department of Surgery, University of Toledo Medical Center, Toledo, OH 43614, USA

**Keywords:** high-grade glioma 1, glioblastoma 2, invasion 3, tumor microtubes 4, formin 5, actin 6

## Abstract

High-grade glioma (HGG, WHO Grade III–IV) accounts for the majority of adult primary malignant brain tumors. Failure of current therapies to target invasive glioma cells partly explains the minimal survival advantages: invasive tumors lack easily-defined surgical margins, and are inherently more chemo- and radioresistant. Much work centers upon Rho GTPase-mediated glioma invasion, yet downstream Rho effector roles are poorly understood and represent potential therapeutic targets. The roles for the mammalian Diaphanous (mDia)-related formin family of Rho effectors have emerged in invasive/metastatic disease. mDias assemble linear F-actin to promote protrusive cytoskeletal structures underlying tumor cell invasion. Small molecule mDia intramimic (IMM) agonists induced mDia functional activities including F-actin polymerization. mDia agonism inhibited polarized migration in Glioblastoma (WHO Grade IV) cells in three-dimensional (3D) in vitro and rat brain slice models. Here, we evaluate whether clinically-relevant high-grade glioma patient-derived neuro-sphere invasion is sensitive to formin agonism. Surgical HGG samples were dissociated, briefly grown as monolayers, and spontaneously formed non-adherent neuro-spheres. IMM treatment dramatically inhibited HGG patient neuro-sphere invasion, both at neuro-sphere embedding and mid-invasion assay, inducing an amoeboid morphology in neuro-sphere edge cells, while inhibiting actin- and tubulin-enriched tumor microtube formation. Thus, mDia agonism effectively disrupts multiple aspects of patient-derived HGG neuro-sphere invasion.

## 1. Introduction

High-grade gliomas (HGG, World Health Organization (WHO) Grade III–IV) account for the majority of adult primary malignant brain tumors. HGGs remain an extremely deadly disease. The most aggressive among these tumors is glioblastoma and its morphological sub-variant gliosarcoma (GBM, and GSM respectively, WHO Grade IV), along with anaplastic astrocytoma (WHO grade III). Patients with GBM face an average survival time of 15 months and a 5-year survival rate of 5.5% [1]. Advances in neurosurgery and anti-tumor technologies have not meaningfully impacted survival, and the standard of care has remained unchanged for over ten years: Cyto-reductive surgery, radiation, and adjuvant temozolomide. In light of these realities, the demand for better treatment options is clear.

One of the greatest clinical challenges in treating adult HGG is the invasive nature of the disease. Early radical attempts to treat GBM with complete hemispherectomy of the affected side eventually resulted in contralateral recurrence [2]. At a single cell level, HGG migrates startlingly great distances away from primary tumors. This migration facilitates resistance to targeted radiation therapy, which is only further complicated by observations that suggest ionizing radiation may promote cell invasion [3,4]. Drugs that target GBM’s angiogenic capacity also increased cellular invasion [5,6]. These factors culminate to present a target that is largely invisible and always on the move.

Previous studies showed that traditional single cell assays have limitations relative to 3D multicellular modeling of HGG invasiveness [7]. In contrast, compact 3D multi-cellular neuro-spheres offer a better in vitro model of the tumor microenvironment characterized by complex cell-cell and cell-matrix interactions [8]. These interactions critically influence chemo-resistance mechanisms and the maintenance of a self-renewing cancer stem-like cell phenotype characteristic of clinical HGG, and notably absent in commercially-available HGG cell lines [8,9]. This more accurate match of experimental model to clinical behavior is essential as we seek to target HGG stem-like cells and their role in primary tumor chemoresistance and invasion.

Tumor cells generate force to propel them into the surrounding brain architecture. Tumor cell invasion is highly dependent on dynamic remodeling of the actin and microtubule cytoskeleton systems. Global cytoskeleton targeting for anti-invasive cancer therapy was validated (i.e., Taxol) in several cancers. In GBM, newly-identified cytoskeleton-enriched structures called tumor microtubes (TMs) were shown to correlate with invasiveness in patient-derived cell line xenografts [10]. Tumor TMs are enriched in F-actin, microtubules, myosin IIa, and the gap junction protein Connexin43. Interestingly, tumor TMs that formed in patient-derived GBM cell lines xenografted into mice were long-lived (days-weeks), and often times extended hundreds of μm in length within the brain microenvironment, thus differentiating them from other cytoskeleton-enriched structures, such as tunneling nanotubes (reviewed in [11]). Targeting the protein machinery in tumor TMs (i.e., Connexin43, GAP-43) by knockdown approaches induced both markedly increased survival, radio- and chemo-sensitivity, through the loss of the GBM tumor TM network [10,12]. Furthermore, tumor TM induction correlated with an increased GBM cell invasion within the brain, possibly through the observed activation of Rho family GTPases [10]. It remains unclear whether targeting the Rho GTPases and their downstream effectors would alter tumor TM formation in GBM.

In GBM, the differential expression of Rho GTPase family members dictates invasive strategies [13]. Rho GTPases are molecular switches that mediate their effects through interactions with downstream effectors. One family of Rho effectors is the mammalian Diaphanous-related formins (mDia1-3), which are encoded by the *DIAPH/DRF* genes. mDia formins are nano-machines that nucleate and elongate linear actin filaments through the activation of conserved Formin Homology 2 domains (FH2). The mDia FH2 domain is flanked by the Dia-autoregulatory domain (DAD) and the Dia-inhibitory domain (DID). DAD and DID intramolecular interactions underlie an autoinhibited conformation that sterically hinders FH2 association with actin monomers. Upon interaction with Rho GTPases, the DAD-DID bonds dissociate, expose the FH2 domain, and promote F-actin nucleation and polymerization [14,15]. mDias also associate with, and stabilize, the microtubule cytoskeleton [16]. We and others validated targeting mDia as an anti-invasive cancer therapy in in vitro GBM, breast, ovarian, and colon human cancer models [7,17,18,19,20,21].

mDia protein function can be pharmacologically manipulated with small molecules. Antagonism has been broadly studied with the small molecule inhibitor of FH2 domain (SMIFH2), which blocks mDia-mediated F-actin assembly [22]. SMIFH2 downregulated p53 expression, and is cardiotoxic to developing zebrafish embryos at concentrations above the IC_50_ suppressing invasion [23,24]. mDia1 knockout was associated with T-cell dysfunction and the development of myelodysplastic syndromes [25,26]. Alternatively, mDia agonism with the small molecules, Intramimic-01 and Intramimic-02 (IMM01 and IMM02), relieved mDia auto-inhibition to induce F-actin polymerization. IMM agonism represents an anti-invasion strategy in cultured GBM cell lines that is superior to SMIFH2 antagonism, by blocking directional and random migration in both spheroids in vitro, and invasion into rat brain slices ex vivo [7]. mDia agonism with IMMs has a significantly lower toxicity threshold in vivo relative to SMIFH2 antagonism [23].

In the current study, we evaluated the efficacy of mDia agonism with IMMs as an effective anti-invasion strategy in a clinically relevant model of patient-derived primary HGG cells, which spontaneously grow as neuro-spheres. mDia formins were enriched in primary HGG tumors. The treatment of patient-derived HGG neuro-spheres, with IMMs, suppressed multiple aspects of tumor cell invasion, including single cell migration from neuro-sphere cores, and directed an amoeboid morphological switch in neuro-sphere edge cells. Interestingly, the formation/maintenance of long actin- and microtubule-enriched pro-invasion tumor TMs was inhibited in response to mDia agonism in neuro-spheres. Collectively these data suggest that IMM-based mDia agonism is a viable strategy for therapeutically targeting multiple mechanisms, underlying adult HGG cellular invasion.

## 2. Results

### 2.1. Patient-Derived Central Nervous System Tumor (CNS) Cell Isolation, Characterization, and Culture

**Figure 1:**
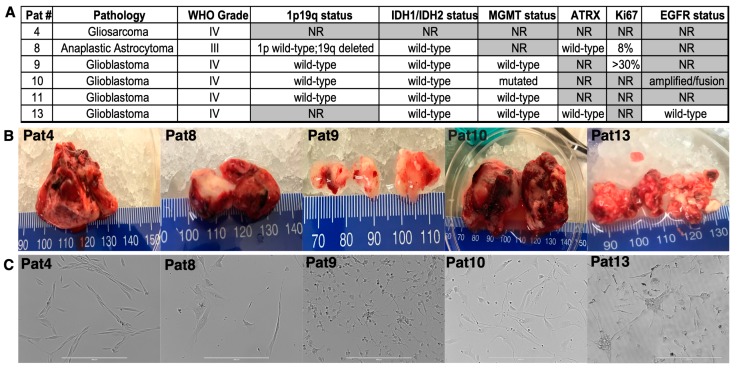
Central Nervous System (CNS) tumor patient samples. (**A**), Pathological and molecular characteristics of tumors from patient surgeries. NR = not reported. (**B**), Gross images of select individual tumors. (**C**), Representative images from select patient cell isolations showing the monolayer cells in 2D-culture. Scale bar = 400 μm.

De-identified suspected high-grade glioma surgical samples were collected and immediately processed to a single cell suspension. CNS tumors were confirmed with pathological analysis (Figure 1A,B). Molecular characterization of tumors was performed, assessing IDH1/IDH2 mutational status (mutations present in a majority of low-grade diffuse gliomas or secondary gliomas and indicative of better outcome and survival [27,28,29]); 1p/19q co-deletion (differentiates oligodendroglioma from astrocytic lineages and predicts greater chemosensitivity [27,29,30]); MGMT methylation (predicts overall survival, due to an increased chemo-sensitivity [29,31]); Ki67 index; and ATRX status (differentiates astrocytoma from oligodendrocyte lineages and used as glioma molecular sub-classification marker [29,32]). The tumor cells from cell suspensions were initially plated upon tissue culture plastic. HGGs including Anaplastic Astrocytoma, Glioblastoma, and the GBM sub-variant Gliosarcoma consistently yielded rigorous long-term cultures (Figure 1C).

In most patient-derived HGGs, cells spontaneously formed 3D neuro-spheres (Figure S1), which detached and were propagated in low-attachment suspension culture (i.e., Patient samples-4 (Pat4), -8 (Pat8), -9 (Pat9), -10 (Pat10) and -13 (Pat13)) for remaining studies.

### 2.2. mDia Formins Are Differentially Expressed in Human HGG Patient Tumors

**Figure 2:**
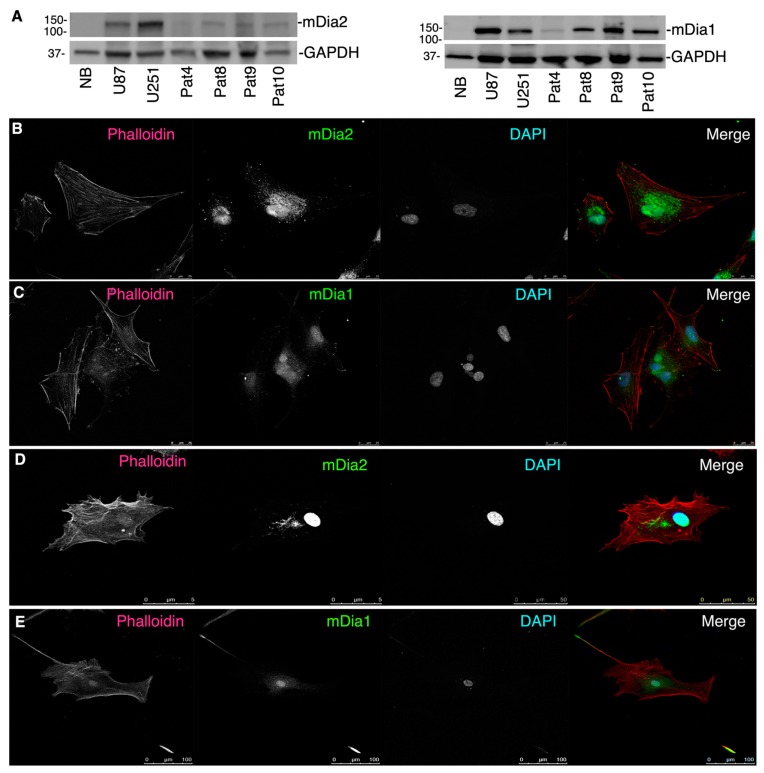
Formin expression in patient tumors cells. (**A**), Western-blots of indicated patient cell line lysates cultured briefly on tissue culture plastic. U87 and U251 cells were used as positive mDia expression controls. (**B**–**E**), IF of patient-derived HGG cells (Pat9, **B**,**C**; Pat10, **D**,**E**) briefly cultured on glass coverslips to visualize mDia1 (green), mDia2 (green), F-actin (phalloidin; red) or nuclei (DAPI; blue).

*DIAPH3* was modestly, yet significantly, upregulated in grades II-IV gliomas, relative to the normal brain and was expressed in U87 and U251 glioma cells [7]. Western-blotting lysates from patient-derived cell monolayers (Pat4, 8, 9, 10) for mDia1 and mDia2 (Figure 2A) showed both proteins expressed to varying degrees. This was confirmed by immunofluorescence (IF) to visualize mDia2 and mDia1 (Figure 2B) in Pat9 (upper panel), and Pat10 (lower panel). mDia2 spatial localization for Pat9 and Pat10 cells was nuclear and perinuclear. mDia1 was both nuclear and diffusely cytoplasmic.

### 2.3. mDia Agonism Inhibits Patient-Derived Neuro-Sphere Invasion

**Figure 3:**
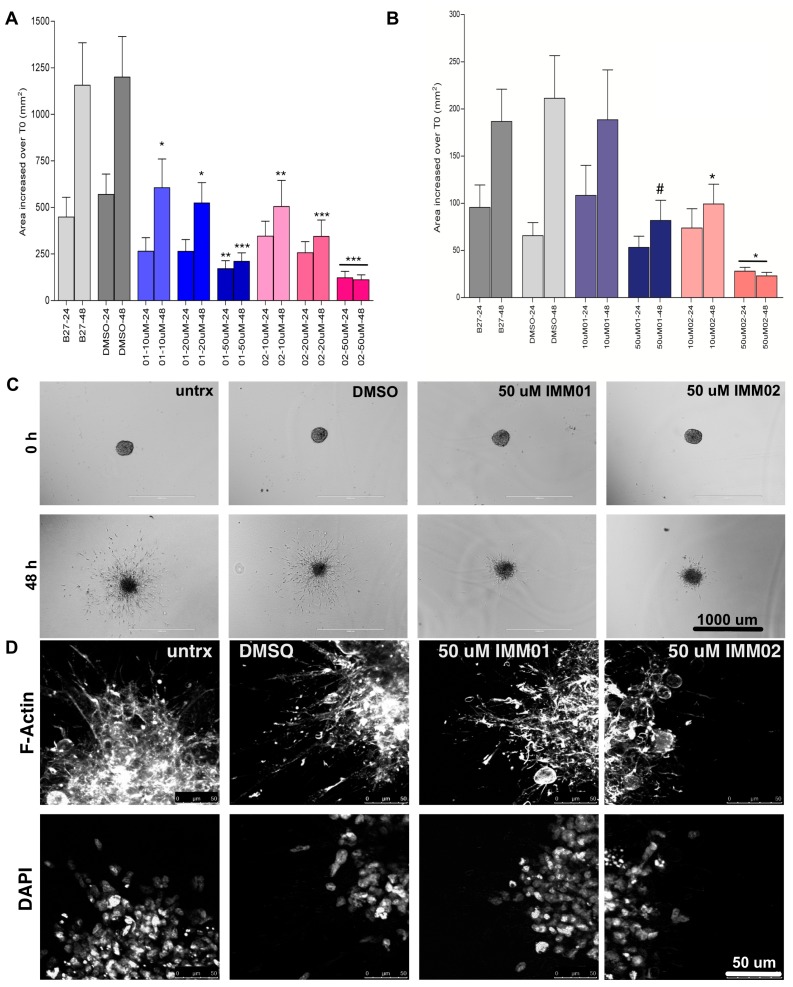
mDia agonists halt high-grade glioma (HGG) patient neuro-sphere invasion. Neuro-spheres were formed and embedded in matrigel. Neuro-sphere areas were measured at embedding (T0), at 24 h and 48 h invasion. (**A**) Change in neuro-sphere invasion graphed as the change in area over T0 for Pat9. N > 12 neuro-spheres/condition over three replicate experiments. p values are relative to the corresponding time point in B27-treated control invasion assays where * *p* < 0.05; ** *p* < 0.02; *** *p* < 0.006. (**B**) Change in neuro-sphere invasion graphed as the change in area T0 for Pat8. N > 12 neuro-spheres/condition over three replicate experiments. *p* values are relative to the corresponding time point in B27-treated control invasion assays where * *p* < 0.0001; # *p* < 0.003. (**C**) Representative images of Pat9 neuro-sphere invasion at 0 and 48 h. Scale bars = 1000 μm. (**D**) Confocal images of fixed Pat9 48 h invasion assay stained for phalloidin and DAPI and showing the neuro-sphere edges. Scale bars = 50 μm.

Pat8 and Pat9 HGG neuro-spheres were embedded in matrigel and allowed to invade. At embedding, the neuro-spheres were treated with 10–50 μM IMM01 or IMM02 and refreshed daily. Images were captured at embedding (T0) and every 24 h. The invasion area was calculated as an increase in area over T0 Pat9 or Pat8 neuro-sphere areas (Figure 3A,B, respectively). Both Pat9 and Pat8 neuro-spheres robustly invaded through 48 h, similar to control vehicle-treated neuro-spheres. However, both IMM01 and IMM02 significantly halted invasions within 24 h relative to controls, and inhibition was maintained through 48 h (Figure 3C,D).

### 2.4. Patient-Derived Neuro-Sphere Invasion Inhibition via mDia Agonism Is Reversible

**Figure 4:**
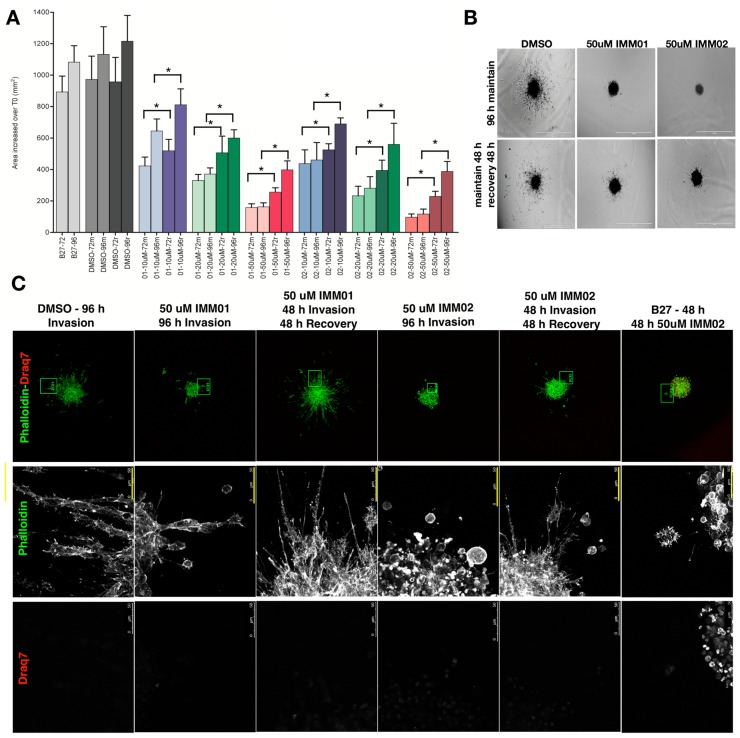
HGG patient neuro-sphere invasion is recovered upon intramimic (IMM) drug washout. (**A**) Invasion assays were performed for Pat9 for 48 h in the presence of indicated drug treatment (DMSO; IMM01; IMM02). Drugs were either maintained thereafter (m) or drug was removed and replaced with media alone (r). Assays were then continued through 96 h. Pat9 neuro-sphere invasion was graphed as change in area relative to neuro-sphere area at the time of embedding (T0). Invasion area was measured 72 or 96 h post-embedding. * *p* < 0.001 by ANOVA. (**B**) Representative neuro-sphere images from Pat9 in (**A**) showing invasion assays at 96 h either maintained in IMMs or recovered in media after 48 h. Scale bars = 1000 μm. (**C**) Pat9 neuro-sphere were incubated with the live-dead cell dye Draq7 (red) and phalloidin (green) at the indicated time points, and with or without drug washout, as in (**A**). In the rightmost panel, control B27-treated neuro-spheres invaded for 48 h, then media was replaced with IMM02-containing media for an additional 48 h. Scale bars = 50 μm.

We previously showed in U87 spheroids that IMM invasion blockade was reversible [7]. We assessed whether IMM anti-invasion effects were reversible (Figure 4A,B). Pat9 neuro-spheres were incubated with vehicle or IMMs for 48 h. Drug washouts were then performed with drug-free media and invasion continued for 48 h. As before, IMM removal released invasion suppression relative to neuro-spheres held in IMMs for 96 h. The live-dead cell dye Draq7 was then used on invading neuro-spheres treated with IMMs. IMM-treated Pat9 neuro-spheres revealed no evidence of apoptosis (as they excluded the dye) when agonists were continuously incubated with neuro-spheres starting at the time of embedding and maintained for 96 h. Unexpectedly, when started at 48 h post-invasion in B27-treated control invading neuro-spheres, IMM treatment for 48 h thereafter induced robust cell death (Figure 4C).

### 2.5. mDia Agonism Halts Formation of F-actin and Tubulin-Enriched Tumor Microtubes (TMs) in Invading HGG Neuro-Spheres

**Figure 5:**
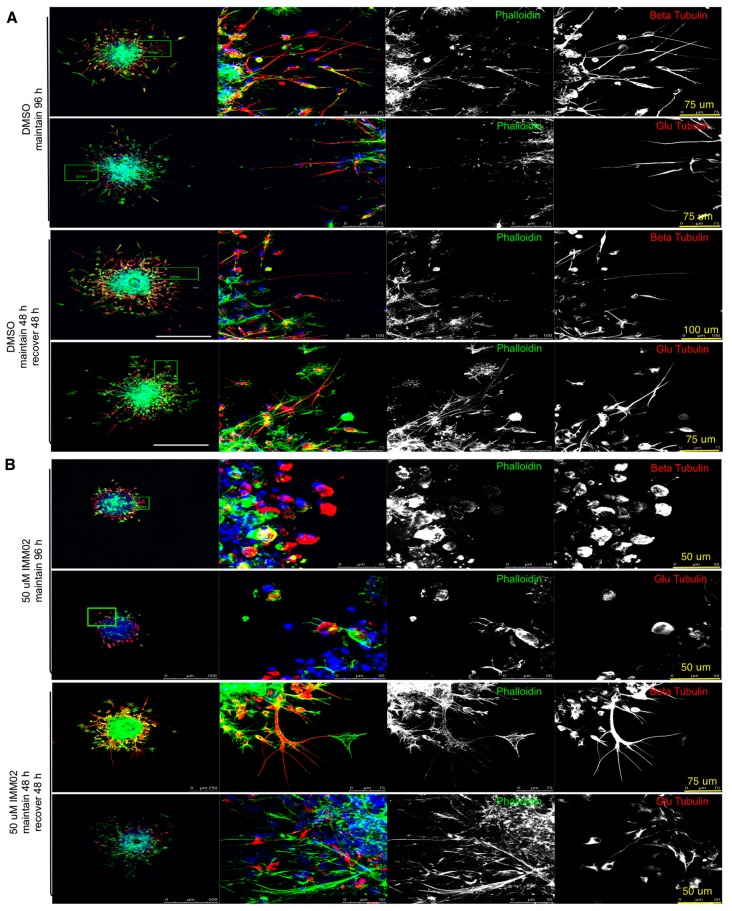
mDia agonism prevents HGG cell cytoskeleton projections. Confocal imaging of fixed Pat9 invasion assays in Figure 4. Imaging was for F-actin (green), β-tubulin or de-tyrosinated α-tubulin (glu-tubulin) (red) at the edge of (**A**) DMSO or (**B**) 50 μM IMM02-treated neuro-spheres. Images are z-stack projections at 96 h of invasion. Treatments were either maintained for 96 h or treatment was removed at 48 h of invasion and replaced with media alone for the remaining 48 h invasion.

IF was performed on fixed Pat9 neuro-spheres treated with or without IMMs for 96 h (Figure 5A,B, respectively). We visualized mDia1, mDia2, F-actin, β-tubulin or de-tyrosinated α-tubulin (glu-tubulin) (to assess stabilized microtubules). Invaded control neuro-spheres had diffuse invasive fronts, with extensive symmetrical invasion away from the central neuro-sphere core. Both mDia1 and mDia2 were diffusely cytoplasmic. The invasive front of control treated neuro-spheres revealed a heterogenous mixture of invading cells of various morphologies (elongated, round, stellate). Some cells were enriched in traditional parallel lamellar actin stress fibers and others with numerous radial F-actin-enriched filopodia-like structures (Figure S2).

Interestingly, in control-treated cells, a portion of invaded cells extended cytoskeleton-enriched protrusions into matrigel (Figure 5A) that measured tens to upwards of several hundreds of microns in length. Control, invaded cells were enriched for F-actin, β-tubulin and glu-tubulin. Specifically, the exaggerated cellular projections in the subset of invaded cells revealed F-actin bundles of varying thicknesses and lengths that ran parallel along the protrusions. Similar enrichment was seen for β- and glu-tubulin along the projection length. Line scanning was performed using Leica software to measure the intensity of the cytoskeletal components along cellular protrusions. Cellular projections were traced on images by drawing lines (green lines, left) along the length of projections from cell bodies to the end of the projection, to measure the fluorescent intensity of a given fluorescent channel, specifically across the (sub-saturated) line. Continuous expressions of a given protein along a protrusion is indicated by a continuous positive fluorescent pixel value greater than 50% of line, whereas discontinuous expression is indicated by <50% continuous positive fluorescent pixel values along the drawn line. Line scans showed F-actin was discontinuous, while β-tubulin and glu-tubulin were robust and continuous along cellular protrusions (Figure S3, representative image). IMM treatment ablated protrusion formation (Figure 5B, upper panel), which then re-formed upon drug washout (Figure 5B, lower panel). The presence of F-actin and glu-tubulin-enriched cellular projections was validated in another HGG patient neuro-sphere culture, Pat13 (Figure 1A, Figure S4), where IMMs inhibited invasion and cellular projection formation.

**Figure 6:**
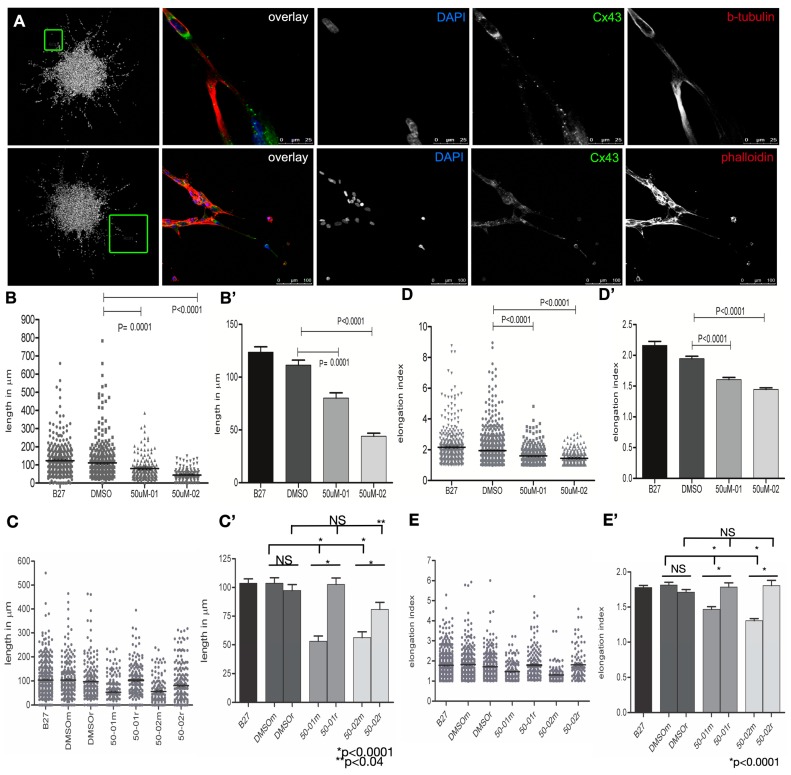
Tumor microtube (TM) shortening and amoeboid cell morphologies in IMM-treated HGG patient-derived neuro-spheres. (**A**) IF for proteins marking TMs (F-actin (red), Connexin43 (green)) and DAPI to denote nuclei in 2 separate Pat8 neuro-spheres invading for 96 h. Left-most boxes indicate areas of magnification shown. Scale bar (upper panel) = 25 μm; (lower panel 100 μm). (**B**,**C**), TM lengths were measured using Metamorph software from cell nuclei to TM ends. TMs measured from N > 196 cells across three replicate experiments. TMs were measured in control (**B**), or (**C**), drug washout experiments using 50 μM IMM01 or IMM02 where m = maintained drug and r = washout. Data are shown as scatter plots (**B**,**C**), or histogram (**B’**,**C’**). (**D**,**E**), TMs were measured in control (**D**) or washout experiments (**E**) using 50 μM IMM01 or IMM02 where m = maintained drug and r = washout. (**D**,**D’**). Cellular morphologies were measured in neuro-sphere edge cells and EI were calculated by dividing cell long by short axes, where EI = 1 is a rounded cell, and E > 1 is an elongated cell. N > 196 cells across three replicate experiments. Data are shown as scatter plots (**D**,**E**), or histogram (**D’**,**E’**).

Cell protrusions were confirmed by IF to robustly express punctate Connexin-43 (Figure 6A). Collectively, our IF revealed these invasive tubulin, F-actin and Connexin-43-enriched cellular protrusions to be consistent with tumor microtubes (TMs). TM length was measured in neuro-sphere edge cells in IMM-treated Pat9 neuro-sphere. TM length was shortened from ~125 μm in control edge cells to ~50–75 μm in IMM02- and IMM01-treated cells (Figure 6B, Figure S5). TM lengths were recovered to control TM lengths upon drug washout (Figure 6C).

By Western-blotting, IMMs caused a modest, yet not statistically significant, decrease in total cellular mDia2 (and mDia1 to a lesser extent) in Pat9 monolayers at 24 h (Figure S6), while total β-tubulin and glu-tubulin were slightly increased (also not statistically significant), as seen previously [17]. To confirm the dependence of TM formation on microtubules, Pat9 neuro-spheres were treated with nocadazole to block microtubule polymerization (Figure S7). Within 24 h, Pat9 invasion and TM formation were halted, indicating a role for microtubules in HGG neuro-sphere invasion.

### 2.6. mDia Agonism Induces Morphological Plasticity in Invading Neuro-Spheres

Finally, in IMM-treated neuro-spheres, a distinct morphological switch occurred (Figure 5 and Figure 6D) in cells surrounding the edges and was accompanied by TM loss. Cells inter-converted to amoeboid morphologies, as confirmed through elongation index (EI) calculations (long/short axis). Amoeboid cells were generally devoid of cortical F-actin, while enriched in β- and glu-tubulin. Amoeboid morphologies were reversed upon drug washout (Figure 6E); elongated cell morphologies were recovered and actin and tubulin-enriched TMs were reformed upon washout (Figure 5). Robust cell motility at the leading invasive edges also resumed (Figure 5B). Thus, the anti-invasion and anti-TM effects of IMM-mediated mDia agonism are reversible in patient-derived neuro-spheres.

## 3. Discussion

The highly invasive nature of adult high-grade gliomas, specifically GBM, represents a significant and deadly clinical challenge that remains unanswered by current therapeutics. mDia formins play important roles in cell motility and invasion in numerous cancer models, including GBM [7,33,34,35]. Here we demonstrate mDia expression in primary high-grade gliomas. Using clinically-relevant neuro-spheres, mDia agonism with IMMs, reversibly inhibits in vitro invasion of primary HGGs. IMMs drive a non-motile amoeboid morphological conversion in cells on the edges of HGG neuro-spheres, and inhibit formation of cytoskeleton and Connexin43-enriched TMs, structures linked to invasiveness and chemoresistance. Thus, mDia agonism effectively disrupted multiple novel aspects of patient-derived HGG neuro-sphere invasion.

IMMs effectively blocked invasion in patient-derived neuro-spheres in multiple *IDH1*-wild-type high-grade glioma patient samples (Figure 3, Figure S3). Inhibition was reversible, suggesting that IMMs as a single agent treatment modality have utility as anti-invasive therapeutics, yet are not cytotoxic, as shown in Draq7 staining. Interestingly, while IMMs were not cytotoxic when applied to neuro-spheres at the start of invasion assays, adding IMMs to already invading neuro-spheres resulted in non-viable neuro-spheres (Figure 4C), perhaps indicating a differential susceptibility in targeting formins to invading single cells. In 2-dimensions, we previously demonstrated neither changes in cell cycle progression, nor increases in apoptosis/multi-nucleation in IMM01/02-treated U251 monolayers [7]. Using monolayer non-transformed NIH3T3s, IMMs impacted cell-cycle progression, while in transformed SW480 colon cancer cells the anti-proliferation effects were less pronounced [17]. However, in 3-dimensions, IMMs were neither toxic to normal rat brain tissues, nor to developing zebrafish embryos [7,23]. IMMs were effective at halting tumor progression in a subset of tumors in animals injected subcutaneously with SW480 colon cancer cells [17], and in ex vivo rat brain slices, IMMs effectively halted GBM invasion without impacting normal brain tissue [7]. It remains uncertain if longer-term IMM treatment of patient-derived neuro-spheres impacts cell cycle, and whether it regulates proliferation to sensitize them to cytotoxic therapeutics [36,37]. We are currently evaluating this hypothesis using patient-derived HGG neuro-spheres with anti-mitotic agents (i.e., temozolomide). Altered mDia function and/or expression sensitized ovarian and prostate cancer cells to chemotherapy [18,38]. Breast cancer patients, with reduced *DIAPH3* expression, were more sensitive to taxol and GBM patients with reduced *DIAPH3* expression showed reduced survival times [38,39]. While these studies focused upon the loss of mDia2 function and/or *DIAPH3* expression, mDia2 agonism could impact tumor cells similarly, altering cells’ ability to drive cytoskeletal dynamics and execute downstream signaling through interacting partners.

Unlike cultured cell lines (i.e., U87, U251), in our hands HGG patient-derived neuro-spheres revealed TMs extending from a sub-set of leading-edge invading cells (Figure 5 and Figure 6). These long protrusions were enriched in stabilized microtubules, F-actin and Connexin-43, distinguishing them as TMs. Targeting actin and microtubule dynamics (i.e., IMMs, nocodazole) effectively halted invasion and inhibited TMs. TMs correlated with poor prognosis and radioresistance in *IDH1* wild-type GBM histopathological patient samples [10]. TMs were correlated with GBM invasion in xenografts, and associated with cell-cell communication within the tumor microenvironment via the propagation of intercellular Ca^2+^ waves [10]. Altering mDia function negatively impacted TM formation in our system; mDia agonism targets the F-actin and microtubule cytoskeleton, both of which appear to have roles within TM formation and/or maintenance. Studies are underway in determining whether mDia2 directs specific cytoskeletal system dynamics within TMs. Furthermore, in light of the presumed stem-like cellular component to our patient-derived neuro-spheres, it would be valuable to evaluate if IMMs effectively target invasion in this important HGG cell subset.

Finally, we unexpectedly observed morphological plasticity in cells on the edges of IMM-treated neuro-spheres (Figure 6). Invasion was weak and amoeboid shaped cells detached from the neuro-sphere itself. mDia inhibition via expression of dominant negative constructs or siRNA, or treatment with SMIFH2 induced amoeboid morphological conversions in a variety of epithelial cancers, including ovarian, prostate, breast and hepatocarcinoma [19,20,21,24,40,41,42]. One possible explanation for this morphological conversion is that, with disrupted mDia dynamic activation, RhoA/ROCK1 contractility/disruption of cell-cell adhesions may be favored [43,44], thereby leading to sustained leading-edge contraction, which drives cellular extrusion. Interestingly, these amoeboid cells do not migrate far from the edge (Figure 5). It is possible that ROCK activation alone is not sufficient to drive motility in these cells, as mDia2 has critical roles in amoeboid-based motility through RhoA/C-directed non-apoptotic bleb retraction and re-establishment of the F-actin cortex [21,40,42]. This leads us to question whether IMMs, combined with ROCK inhibition, would be a most effective anti-invasion strategy in HGG patient-derived neuro-spheres. Studies are currently underway assessing this hypothesis.

## 4. Materials and Methods

### 4.1. Cell Culture

U87 cells were a kind gift from Dr. Willaim Maltese and Jean Overmeyer (University of Toledo). U251 cells were a kind gift from Marthe Howard (University of Toledo). GBM cells were maintained in DMEM (HyClone, Logan, UT, USA) with 10% FBS (vol/vol) (Atlantic Biological, Estates, FL, USA), 100 U/mL penicillin and 100 mg/mL streptomycin (Gibco, Waltam, MA, USA) in a 37 °C humidified incubator with 5% CO_2_.

### 4.2. Cell Isolation and Cryofreezing

All subjects gave their informed consent before they participated in the study. The study was conducted in accordance with the declaration of Helsinki, and the protocol was approved by the University of Toledo Institutional Review Board (IRB#201913). De-identified surgical samples were collected from the University of Toledo Medical Center or ProMedica Toledo Hospital using a joint consent form. Resected tumors were transported in PBS on ice. Single-cell isolation was performed as described with minor modifications [45,46]. Briefly, tumors were washed with D-PBS and photographed with an iPhone7 camera (Cupertino, CA, USA). The tumors were minced with surgical scalpels. For cell isolation, a portion of minced tumors were placed in 15 mLs of 0.05% trypsin (Gibco) and rotated at 37 °C for at least 45 min. Tumors were triturated, and tissue returned to 37 °C with rotation. Trypsin was neutralized in equal volumes of Neural Basal Media/10% FBS. After 5 min room-temperature (RT) incubation, cells were centrifuged at 1000 rpm for 5 min and resuspended in 10 mLs of ice-cold Red Blood Cell Lysis Buffer (0.15 M NH_4_Cl, 10 mM NaHCO_3_ and 0.1 mM EDTA). This reaction was neutralized with equal volumes ice-cold DPBS (HyClone). Cells were centrifuged at 250× *g* for 5 min, resuspended in Neural Basal Media (Gibco) supplemented with 1× B27 (Gibco), 20 ng/mL bFGF and EGF (Peprotech, Rocky Hill, NJ, USA), 1× Sodium Pyruvate, 1× GlutaMax, and 1× Anti-Anti (Gibco). Cells were strained through 70 μM strainers (Fisher Scientific) and plated into 6-well tissue culture plates (USA Scientific, Ocala, FL, USA). Media was changed after 24 h.

For cryo-freezing and cell recovery [47,48], minced tissue was placed in cryotubes (Greiner, Monroe, NC, USA) with full media and 10% DMSO (Fisher Scientific, Hampton, NH, USA) and placed in a freezing container (Nalgene, Rochester, NY, USA) that lowers the temperature 1°/min. Tissues were placed at −80 °C overnight, and moved to −150 °C.

### 4.3. Neuro-Sphere Formation and Culture

Neuro-spheres formed spontaneously in isolated patient sample monolayer cells. Once the spheres detached from the monolayers, they were collected using wide-orifice pipette tips and moved to poly-HEMA (Sigma, St. Louis, MO, USA) coated U-bottom 96-well plates. Neuro-spheres were used upon reaching 200–250 μm diameter. Once neuro-spheres reached 350 μm, they were dissociated and re-plated in poly-HEMA U-bottom plates at 2000 cells/well.

### 4.4. Invasion Assays

The 3-dimensional invasion assays were as described in [7,20]. Briefly, a thin layer of 5 mg/mL GFR matrigel (Corning, Tewksbury, MA, USA) was placed in 8-well chamber glasses (Lab-tek, Rochester, NY, USA). Neuro-spheres were added and topped with thin-layer matrigel. Matrigel polymerized for 45 min at 37 °C before addition of 250 μL of media with IMMs [7,17]. The invasions were imaged at time zero (T0) and every 24 h for experimental durations. Invaded spheroids/spontaneous neuro-spheres were imaged using an EVOS inverted microscope with an Olympus 4× UplanFL N0.13 PhP objective lens (Center Valley, PA, USA). All measurements were completed with MetaMorph software (Molecular Devices, San Jose, CA, USA).

### 4.5. Immunofluorescence (IF)

Draq7 (Abcam, Cambridge, MA, USA) was loaded into neuro-spheres as described [49]. For 3D IF, neuro-spheres were stained as described, with modifications [49]. Invasions were fixed in 4% paraformaldehyde/PBS for 20 min at RT. Chambers were permeabilized with 0.5% Triton X-100/PBS (Sigma) for 60 min at RT. Chambers were washed in PBS-T (PBS with 0.1% Triton X-100) and blocked in 3% BSA for 1 h at RT. mDia2 and mDia1 (1:100) or Connexin-43 (1:200) (Proteintech, Rosemont, IL, USA), β-Tubulin (1:100) (Sigma), Glu-Tubulin (1:100) (Millipore Sigma, Burlington, VT, USA) antibodies were incubated at 4 °C for 48–72 h. Invasions were washed with PBS-T before adding AlexaFluor 2° antibodies (1:200–500), AlexaFluor Phalloidin (1:100), or DAPI (1:50) (Molecular Probes, Eugene, OR, USA) for 24–48 h at 4 °C. The wells were imaged on a Leica TCS SP5 multiphoton laser-scanning confocal microscope. Optical slices of 2.5 μm were acquired in Z-stacks using a Leica 10× Pl Apo CS Dry 0.40NA objective (Buffalo Grove, IL, USA).

For 2D IF, the cells were processed as described [20,21]. The cells were plated on coverslips, fixed in 4% paraformaldehyde/PBS for 5 min at RT, permeabilized with 0.2% Triton X-100 for 20 min before incubating with antibodies: mDia1, mDia2, β-Tubulin (1:200) (Sigma), and Glu-Tubulin (1:200) (Millipore) were incubated ON at 4 °C. Coverslips were incubated ON at 4 °C with (1:500) AlexaFluor 2° antibodies, (1:500) AlexaFluor phalloidin and (1:100) DAPI. The coverslips were washed in PBS, followed by deionized-water and were mounted to slides with Fluoromount-G (Southern Biotech, Birmingham, AL, USA). The coverslips were imaged on a Leica TCS SP5 multiphoton laser-scanning confocal microscope, using 2.5 μm optical slices assembled into Z-stacks using a Leica 40× HCX Pl Apo CS Oil 1.25–0.75NA objective or a 63× HCX Pl Apo CS Oil 1.4-0.6NA objective.

### 4.6. Western-Blotting

Confluent cell monolayers were lysed (0.5M Tris-HCL pH 6.8, glycerol, 10% (*w*/*v*) SDS, and bromophenol blue supplemented with dithiotheritol (DTT). Lysates were quantified by BioRad DC Protein Assay, loaded into a 4–20% mini-protean TGX gel (BioRad, Hercules, CA, USA) and transferred to PVDF membranes (BioRad) (Trans Blot Turbo System (BioRad)) then blocked in 5% non-fat dry milk. mDia1, mDia2, Glu-tubulin, β-tubulin and GAPDH (Proteintech) antibodies were incubated ON at 4 °C. The blots were washed with TBST and peroxidase-conjugated 2° antibodies (Jackson ImmunoResearch, West Grove, PA, USA) were incubated at RT for 90 min. The blots were imaged on a Syngene Western Blot Imager (Frederick, MD, USA).

### 4.7. Statistical Analysis

Analyses were performed using GraphPad software (San Diego, CA, USA). ANOVA or Students *t*-tests were performed to assess statistical significance, as indicated. A *p* value < 0.05 was statistically significant.

## 5. Conclusions

In conclusion, we discovered the roles of mDia2 in regulating the dynamic cytoskeleton, in support of TM-directed, invasive, adult high-grade, glioma patient-derived neuro-spheres. IMM agonists effectively halt invasion, while suppressing TM formation and eliciting a distinct amoeboid morphological transition in tumor cells. This study brings us closer to understanding the clinically-relevant role mDia2 plays in HGG invasion.

## Supplementary Materials

The following are available online at https://www.mdpi.com/2072-6694/11/3/392/s1. Figure S1: Patient-derived HGG neuro-spheres spontaneously form, Figure S2: Actin enrichment in migrating Pat9 neuro-sphere cells, Figure S3: Cytoskeleton-associated protein expression in HGG neuro-sphere projections, Figure S4: mDia agonism prevents HGG cell cytoskeleton projections, Figure S5: TM measurements in HGG patient neuro-spheres, Figure S6: mDia and cytoskeleton protein expression in IMM-treated patient neuro-spheres, Figure S7: Requirement for microtubule polymerization in neuro-sphere invasion and TM formation.
