# Supplementary material for: Targeting the mDia Formin-Assembled Cytoskeleton Is an Effective Anti-Invasion Strategy in Adult High-Grade Glioma Patient-Derived Neurospheres
Source: Cancers (Basel). 2019 Mar 20;11(3):392. doi: 10.3390/cancers11030392 (PMC6468841; doi:10.3390/cancers11030392)
Supplement: Supplementary file 1 [file cancers-11-00392-s001.pdf]

Supplementary Materials

Targeting the mDia Formin-Assembled Cytoskeleton Is an Effective Anti-Invasion Strategy in Adult High-Grade Glioma Patient-Derived Neurospheres

Krista M. Pettee, Kathryn N. Becker, Arthur S. Alberts, Kevin A. Reinard, Jason L. Schroeder and Kathryn M. Eisenmann

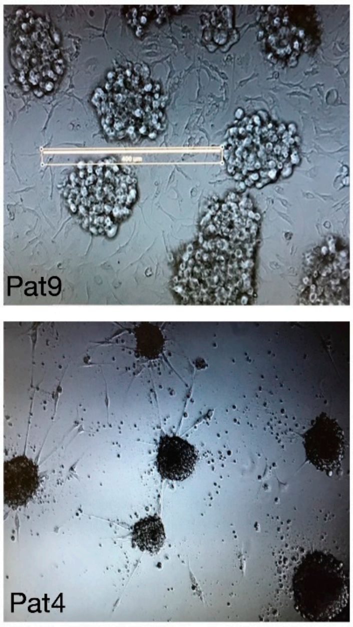

Figure S1. Patient-derived HGG neuro-spheres spontaneously form. Neuro-spheres formed from Pat9 (upper) and Pat4 (lower) primary cultures within days of plating as monolayers. Neuro-spheres were collected and propagated in suspension culture. Magnification = 10×.

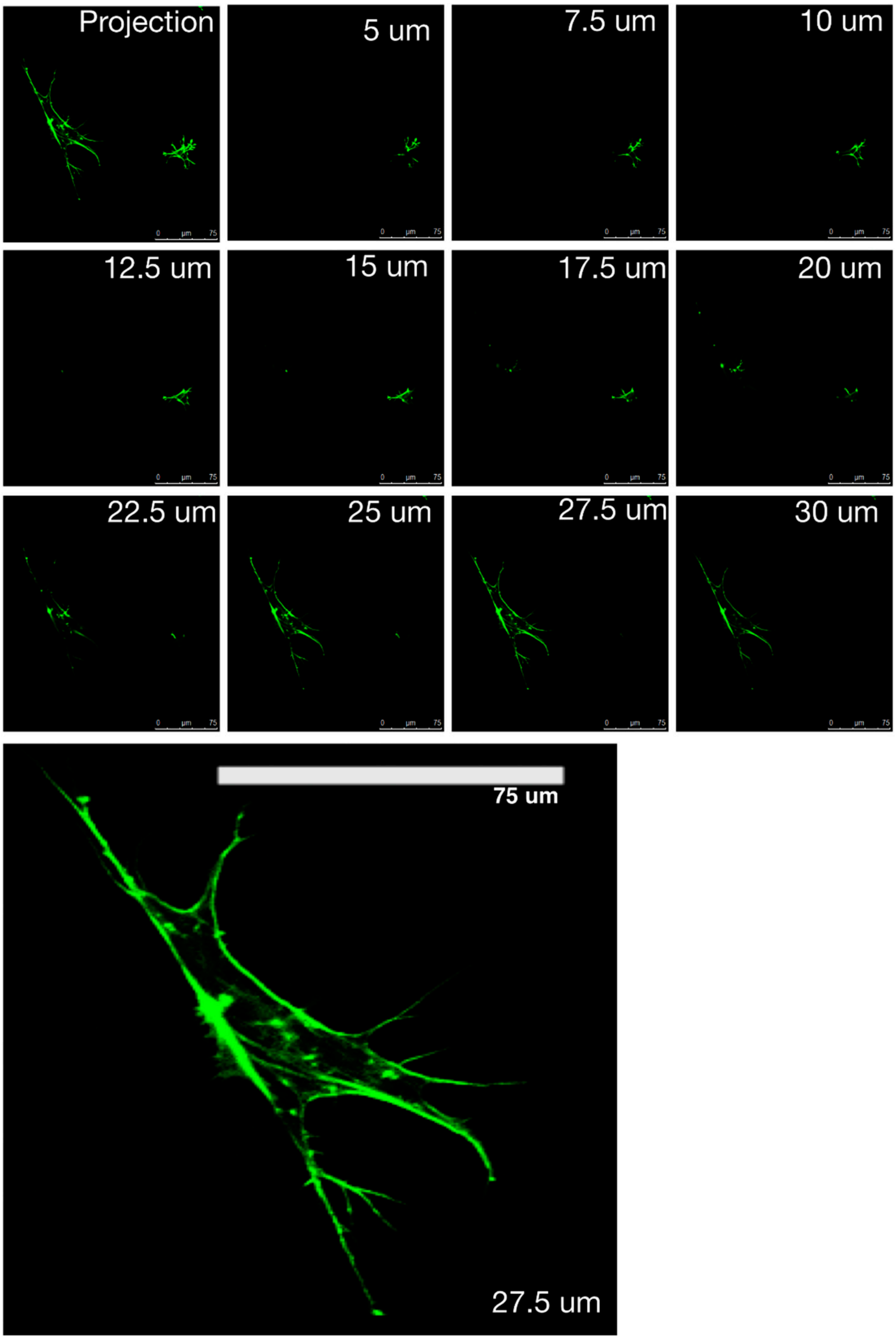

Figure S2. Actin enrichment in migrating Pat9 neuro-sphere cells. Pat9 neuro-spheres were allowed to invade for 96 h and were stained for F-actin (phalloidin) to visualize the F-actin cytoskeleton. Upper left is Z-projection from confocal stack, while remaining images are serial optical slices of the field. Left image is enlargement of the 27.5 μm slice to show details of cytoskeletal projections and stress fibers.

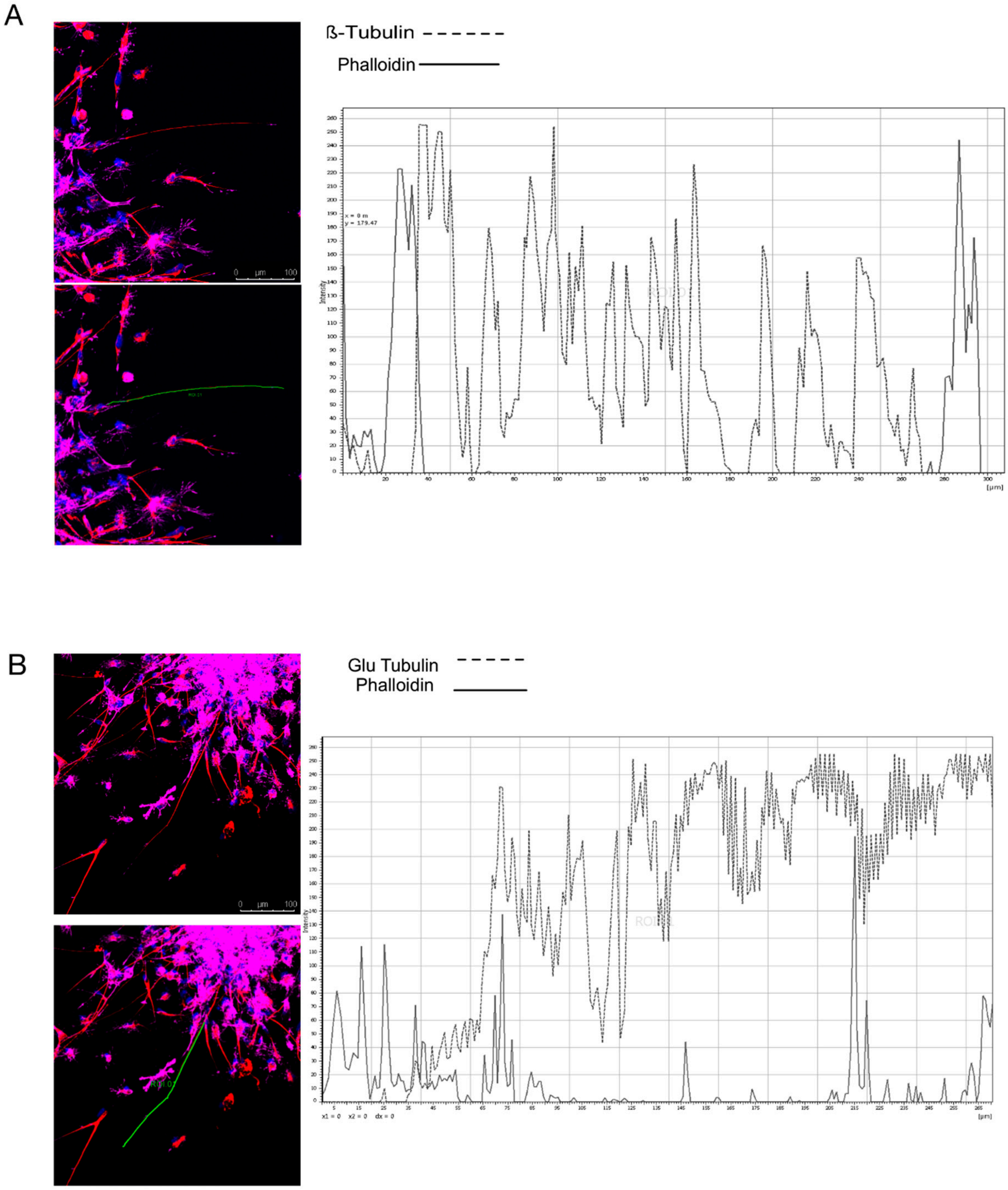

Figure S3. Cytoskeleton-associated protein expression in HGG neuro-sphere projections. β-tubulin (**A**), glu-tubulin (**B**), and F-actin (**A**,**B**) expression in patient neuro-spheres were assessed. Invaded Pat9 neuro-spheres were fixed after 96 h invasion. Immunofluorescence was performed (left) for indicated proteins and imaged by confocal microscopy using a 63× objective. TMs were measured on images using Leica software by drawing lines (green lines, left) along the length of TMs from cell bodies to the end of the TMs to measure the fluorescent intensity of a given fluorescent channel across the line. Histograms of the noted line scan (right) were created for each channel to graph the fluorescent intensities across the length of the TM. "Continuous" expression along a protrusion refers to positive continuous fluorescent value along greater than 50% of line, whereas discontinuous expression is indicated by <50% continuous positive fluorescent values along the drawn line.

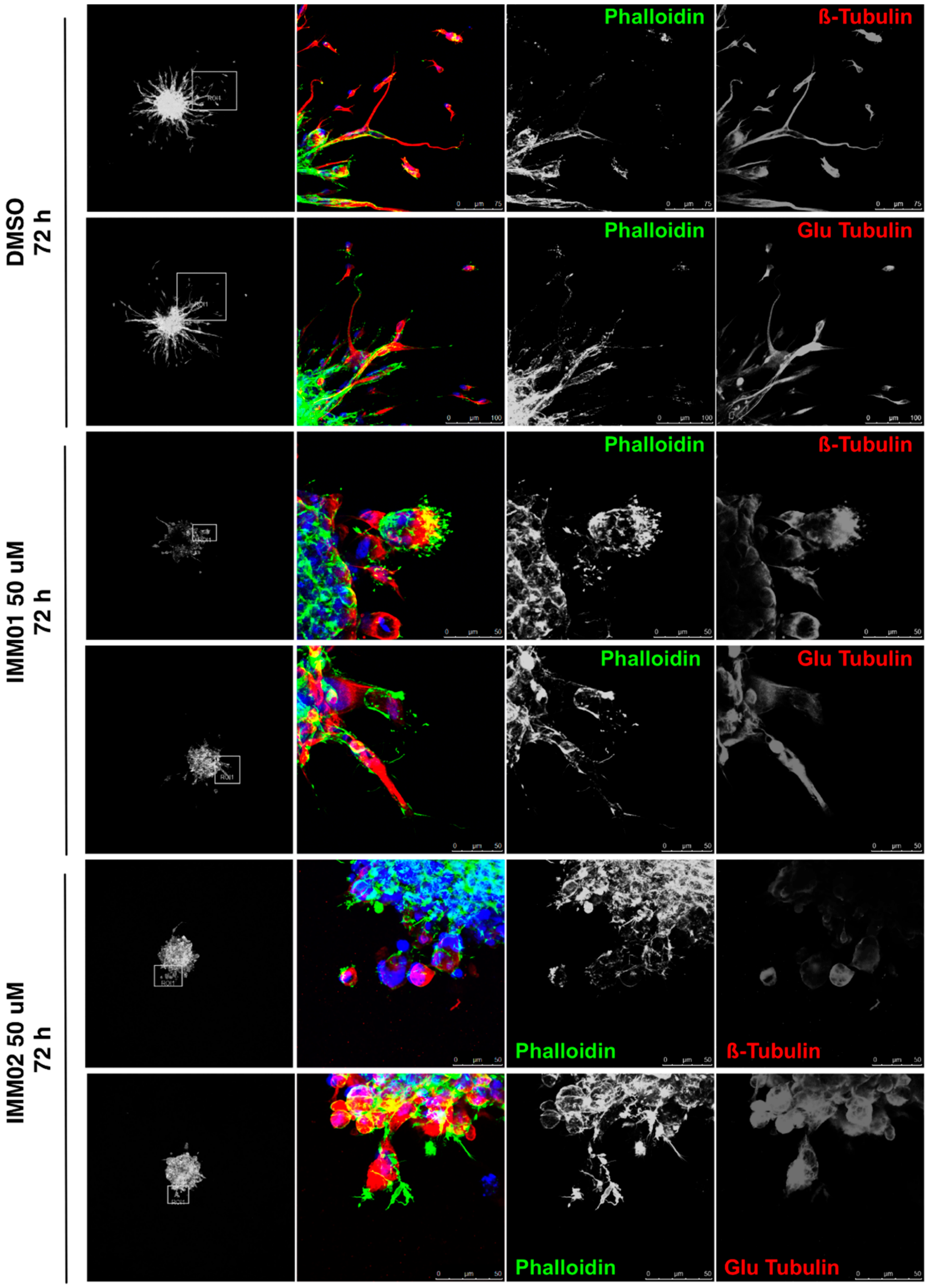

Figure S4. mDia agonism prevents HGG cell cytoskeleton projections. Confocal imaging of fixed Pat13 invasion assays for F-actin, β-tubulin or glu-tubulin at the edge of DMSO (top) or 50 µM IMM01- (middle) or IMM02 (bottom)-treated neuro-spheres. Images are z-stack projections at 72 h invasion.

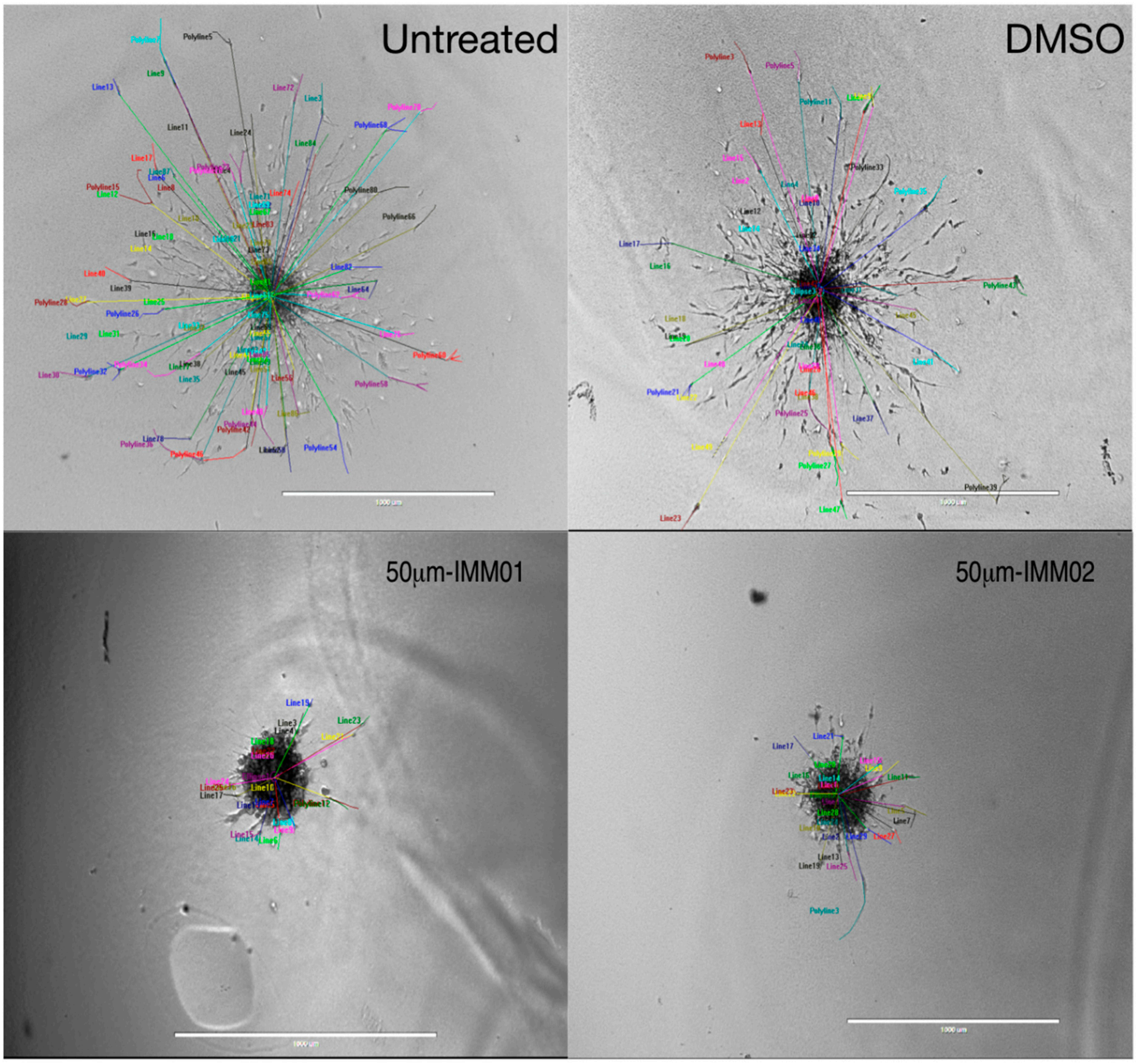

Figure S5. TM measurements in HGG patient neuro-spheres. Pat9 neuro-spheres invaded for 96 h in matrigel in presence of DMSO, 50 µM IMM01, or 50 µM IMM02. TMs were measured using Metamorph software by drawing lines (colored lines) along the length of TMs from cell bodies to the end of the tumor TM using brightfield images.

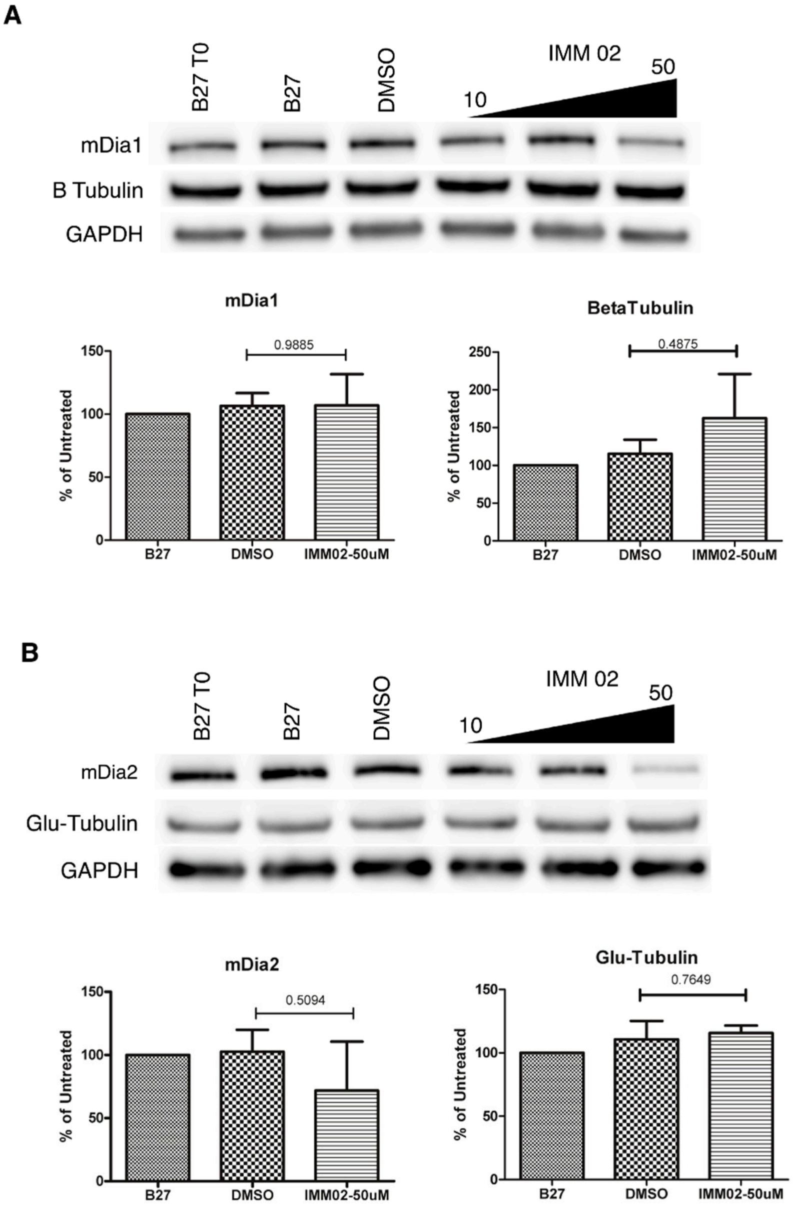

Figure S6. mDia and cytoskeleton protein expression in IMM-treated patient neuro-spheres. Pat9 monolayers were grown in presence or absence of 10, 20 or 50 μM IMM02, B27 control or DMSO vehicle control for 24 h, or B27 (at T0). Western blotting was performed on cell lysates for mDia1, β-tubulin, and GAPDH as a loading control (**A**), or mDia2, glu-tubulin, or GAPDH (**B**). Densitometry was performed on indicated proteins (lower panels), normalizing expression to GAPDH. The experiment was performed in triplicate and standard errors for the averages are shown. p values are indicated.

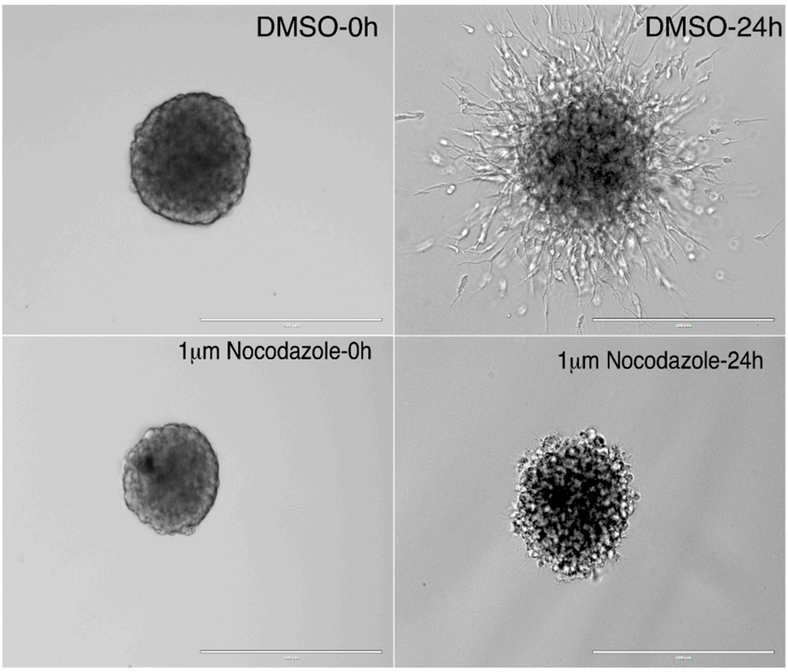

Figure S7. Requirement for microtubule polymerization in neuro-sphere invasion and TM formation. Pat9 neuro-spheres were embedded in matrigel and treated with DMSO or 1 μM nocodazole for 24 h. Images were acquired using brightfield microscopy.

© 2019 by the authors. Licensee MDPI, Basel, Switzerland. This article is an open access article distributed under the terms and conditions of the Creative Commons Attribution (CC BY) license (http://creativecommons.org/licenses/by/4.0/).
